# Hot Fusion: An Efficient Method to Clone Multiple DNA Fragments as Well as Inverted Repeats without Ligase

**DOI:** 10.1371/journal.pone.0115318

**Published:** 2014-12-31

**Authors:** Changlin Fu, William P. Donovan, Olga Shikapwashya-Hasser, Xudong Ye, Robert H. Cole

**Affiliations:** Biotechnology, Monsanto Company, St. Louis, Missouri, United States of America; Tsinghua University, China

## Abstract

Molecular cloning is utilized in nearly every facet of biological and medical research. We have developed a method, termed Hot Fusion, to efficiently clone one or multiple DNA fragments into plasmid vectors without the use of ligase. The method is directional, produces seamless junctions and is not dependent on the availability of restriction sites for inserts. Fragments are assembled based on shared homology regions of 17–30 bp at the junctions, which greatly simplifies the construct design. Hot Fusion is carried out in a one-step, single tube reaction at 50°C for one hour followed by cooling to room temperature. In addition to its utility for multi-fragment assembly Hot Fusion provides a highly efficient method for cloning DNA fragments containing inverted repeats for applications such as RNAi. The overall cloning efficiency is in the order of 90–95%.

## Introduction

Molecular cloning is a daily occurrence in modern molecular biology to study gene expression and element function. As a growing number of genes and regulatory element sequences (such as promoters and terminators) become available, there is an increasing need for rapid cloning to determine their functions. To facilitate high throughput cloning the method of choice should be simple, efficient, robust and compatible with any DNA sequence, while providing directionality. Many methodologies have been developed for directional cloning and multi-gene assembly, including Gateway recombinational cloning [1], [2], [3], [4], [5], In-fusion [6], [7], [8], Cold Fusion [9], Cold Fusion like cloning [10], [11], [12], T4 DNA polymerase-based Ligase Independent Cloning (LIC) [13], [14], [15], [16], uracil-DNA glycosylase (UDG)-based LIC [17], SfiI-based assembly [18], and a number of Type IIs enzyme-based assembly strategies [19], [20], [21], [22], [23], [24]. Among these methods, Gateway recombination cloning has been widely used for cloning a broad range of genes and elements in part because cloning is not dependant on restriction sites. One limitation of the Gateway system is that it requires the construction of entry vectors [1], which takes time and has associated costs, as well as placing limitations on the number of fragments to be cloned. Two cloning systems, referred to as Golden Gate [21] and GoldenBraid [24] have been used for multi-gene assembly and domain shuffling. However they are limited by its dependence on both ligase and Type IIs restriction enzymes for cloning. Type IIs restriction sites are often less than 7 bp long and are frequently present within DNA sequences to be cloned, which places limitations when cloning of multiple DNA fragments. Gibson et al. have described a method that uses T5 exonuclease, DNA polymerase and Taq ligase to simultaneously clone multiple DNA fragments without reliance on restriction sites [25], [26]. Recently a commercially available kit (NEB) has been developed based on the Gibson technology.

In our work there is a strong need for high-throughput cloning to support gene discovery. In an effort to enhance the efficiency of cloning, flexibility and ease of construct design, while meeting the growing demands of our gene discovery pipeline, we have developed a method referred to as Hot Fusion. Hot Fusion is a high throughput cloning method that is independent of restriction sites for inserts and does not require DNA ligase. Hot fusion has advantage over the Gibson et al. assembly method by eliminating background due to self-ligation and eliminating the requirement for vector purification. Hot Fusion performs well for single fragment cloning, multi-fragment assembly and RNAi constructs containing inverted repeats with a cloning efficiency of roughly 90–95%.

## Materials and Methods

### Bacterial strains and growth conditions

Plasmids were introduced into *Escherichia coli* by either electroporation or chemical transformation. Cells were propagated in LB (Luria-Bertani) or TB (Terrific Broth) medium containing the appropriate antibiotics (50 µg/ml for spectinomycin, 20 ug/ml for kanamycin and 60 ul/ml for carbenicillin) plus X-gal (100 ug/ml) for blue-white selection whenever necessary.

### Enzymes and reagents

Restriction enzymes were purchased from New England Biolabs (Beverly, MA). KOD Hot Start DNA polymerase was purchased from Novagen (San Diego, CA). Phusion Hot Start DNA polymerases and GeneRuler 1 kb Plus DNA Ladder were purchased from Thermo Scientific (Waltham, MA). DNA and PCR product purification kits were purchased from Qiagen (Germany) and Zymo Research Corp (Orange, CA). Most chemicals and antibiotics were purchased from Sigma-Aldrich (St. Louis, MO). pSV-β-Gal vector containing *lacZ* was purchased from Promega (Madison, WI).

### Primer design and sequence analysis

DNA analysis and primers used for PCR and sequencing were done by DNA Star software (DNASTAR Inc., Madison, WI) or in some cases manually. Primer pairs used for Hot Fusion were designed with gene specific sequences along with portion of the vector sequences or portion of the junction regions for multiple fragment assembly, each with a 17–30 bp overlap.

### PCR and PCR screening

PCR was usually performed in a 25 µl total volume reaction containing 2.5 µl of 10× PCR buffer, 1.0 µl of dNTPs (10 mM), 1.0 µl of 5 primer oligo (10 µM), 1.0 µl of 3 primer oligo (10 µM), 0.5 µl KOD Hot Start DNA Polymerase (2 u/µl), 1 µl of DNA template [for plasmid DNA (∼10 ng/µl), and for cDNA or gDNA (∼50 ng/µl)], and sterile water to 25 µl. The PCR cycling program was as follows: 1^st^ step: 94°C for 3 min; 2^nd^ step: 94°C for 20 sec; 3^rd^ step: 58°C for 15 sec; 4^th^ step: 72°C for 2–3 min (1 min/kb); 5^th^ step: go back to step 2 for 24 times; 6^th^ step: 72°C for 5 min and 7^th^ step: 10°C on hold. The PCR products were run on agarose gels (0.8–1.2%) and purified by Qiagen columns when necessary.

A number of colonies were picked from each plate (for each construct). Each well of a 96-deep well plate (Qiagen, Germany) containing 200 µl of TB medium plus appropriate antibiotic or 4 ml of TB plus antibiotic in a 14-ml Falcon tube (Becton Dickinson, NJ) was inoculated with an individual colony, and incubated in a 37°C shaker (250 RPM) overnight.

Colonies were screened by cultured-cell PCR with either gene-specific or universal primers (located on the backbone vector). The cultured-cell PCR was performed for 25 cycles with the same PCR program described above.

### DNA isolation and gene synthesis

Plant genomic DNA and plasmid DNA was isolated using Qiagen DNA Isolation Kit according to the Manufacture's instructions. Purified DNA was used for restriction enzyme digestion analysis and sent for sequencing in house. Some DNA fragments were synthesized through the gBlocks of Integrated DNA Technologies (Coralville, Iowa).

### Vector preparation

Vector was digested by restriction enzyme(s) and heat-inactivated at 65°C or 80°C for 20 minutes dependent on enzymes used. If necessary (when the enzyme was not heat-inactivated), enzyme-digested vector was also run on 1.0% agarose gel and purified by Qiagen column or Zymoclean kit.

### Preparation of 5× pre-assembly buffer for Hot Fusion

One ml of 5× pre-assembly buffer (0.5 M Tris pH 7.5, 50 mM MgCl_2_, 1 mM each dNTP, 50 mM DTT, 25% PEG-8000) was prepared by mixing 0.5 ml of 1 M Tris pH 7.5, 50 µl of 1 M MgCl_2_, 100 µl of 10 mM dNTP mix (NEB), 50 µl of 1 M DTT, 250 mg PEG-8000, 150 µl of dH_2_O. Buffer was stored at −20°C.

### Preparation of Hot Fusion buffer

Four-hundred µl of 2× Hot Fusion buffer was prepared by mixing 160 µl of 5× pre-assembly buffer, 0.3 µl of 10 u/µl T5 exonuclease (Epicentre, Madison, WI), 10 µl of 2 u/µl Phusion polymerase, 230 µl of dH_2_O. The final concentration of 2× Hot Fusion buffer contains 0.0075 u/µl T5 exonuclease, 0.05 u/µl Phusion Hot Start DNA polymerase. The buffer was transferred in 10 µl aliquots to 0.2 ml PCR tubes or 200–400 µl aliquots in 1.5 ml microcentrifuge tubes (for multiple uses) and stored at −20°C with no more than 10 times freezer thaw cycles.

### Set up of Hot Fusion reaction

One µl of a linearized vector (30–50 ng/µl) and 2–3 µl of PCR insert (30–100 ng/µl) were added to a PCR microtubes containing 10 µl of 2× Hot Fusion Buffer. Distilled water was added to the tube for a final volume of 20 µl. For multiple fragment assembly (3–4 fragments), 1 µl of each insert (20–50 ng/µl) was used. Tubes were incubated in a thermocycler for 1 hour at 50°C, then slowly ramped down to 20°C in 5 minutes (0.1°C per second), and held at 10°C or as indicated in the Figure's legend. The Hot Fusion reaction was used for transformation or stored at −20°C if not used immediately.

### Electroporation

One µl of Hot Fusion reaction was mixed with 20 µl of ElectroMax DH10B cells (Invitrogen) in a 1.5 ml centrifuge tube. Cells were transferred to a 0.1 cm electrocuvette (BioRad), and electroporated at 1.8 kv voltage setting. Electroporated cells were transferred to a 1.5 ml centrifuge tube, and mixed with 300 µl of SOC medium (Invitrogen) and incubated for 1 hour at 37°C. Between 10 µl and 150 µl of cells were plated onto LB plates containing appropriate antibiotics, and X-gal when needed. Plates were then incubated overnight at 37°C.

## Results

### Development of Hot Fusion cloning technology

**Figure 1:**
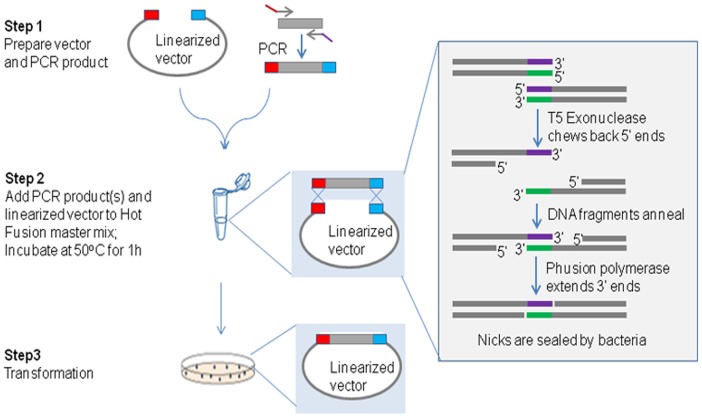
A diagram of the Hot Fusion process for single fragment cloning. Red and blue boxes on the vector and PCR product indicate the overlapping sequences (17–30 bp). T5 exonuclease removes nucleotides from the 5′ to 3′ end of double strand DNA molecules. Phusion DNA polymerase fills in gaps that are over-generated by T5 exonuclease. Annealed fragments are transformed into *E. coli* and the nick sites in the nucleotide chain are repaired by *E. coli.*

**Figure 2:**
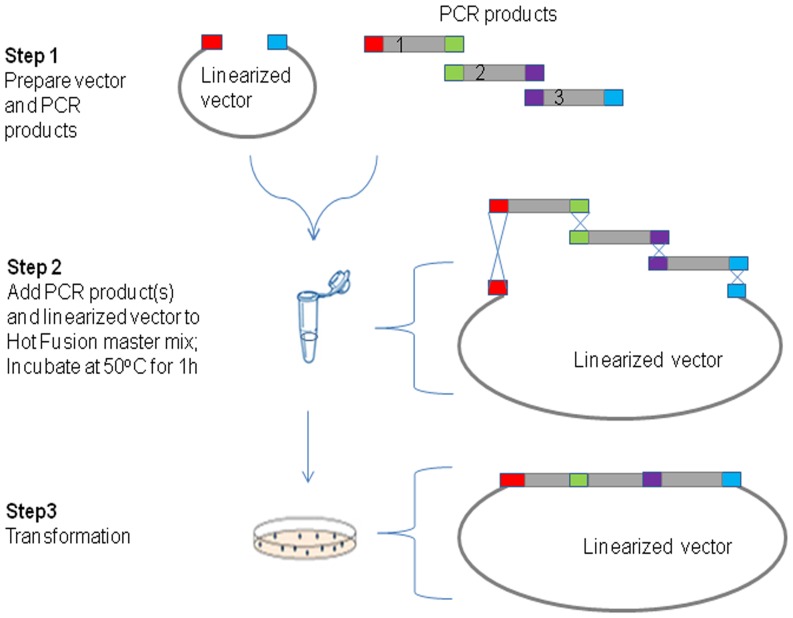
A diagram of the Hot Fusion process for multi-fragment assembly. Red, green, purple and blue boxes on the vector and PCR products indicate overlapping sequences (25–30 bp).

To streamline our cloning platforms and increase cloning efficiency, especially for multi-fragment and complex constructs a simple, robust and automatable cloning method has been developed which we term Hot Fusion. The steps and molecular reactions involved in Hot Fusion are outlined in Fig. 1 for single fragment cloning and in Fig. 2 for multi-fragment assembly. The technique relies on the ordered assembly of DNA fragments based on 17–30 bp overlapping sequences between adjacent fragments (Table 1). Since assembly is based on unique overlapping sequences, multiple fragments can be assembled in a defined order in a single tube. The single tube assembly is carried out at 50°C for one hour. Based on the design of the primers used to generate the DNA fragments, the PCR products are assembled in the pre-determined order and orientation. Following the annealing step, Phusion Hot Start DNA polymerase fills in the gaps generated by T5 exonuclease. The annealed fragments are transformed into *E. coli* and nicks are repaired *in vivo*. This is a highly efficient process which does not require *in vitro* ligation or the use of a ligase in the reaction mixture.

**Table 1:** Examples of the oligo primers used in this study.

Primer name	Sequences (5' ->3')	Application
Mon-For1	**GCGCTGTGCCTGTTGCG**AT CGCACCGGATCTCTATAATCTCGCGCAACC	single fragment cloning
Mon-Rev1	**GCGCGTGTTCTGCTGGCG**AT CGCGGAATAATAGCGAGAACAGAGAAATAGC	
ICD-For1	**AAAAGAGATGGAGGAAGAAG**NNNN(24)	Single fragment cloning
ICD-Rev1	**CTGAATTCTGAGAGCAAG**NNNN(24)	
lacZ-Frag1-For1(1)	**GGTACCTCAGCGCTGTGCCTGTTGCG** ATACCGGATCTCTATAATCTCGCGCAACC	Multi-fragment cloning
lacZ-Frag1-Rev1(420)	**CGCAACTGTTGGGAAGGGCGATCGGTG**	
lacZ-Frag2-For2(394)	**CACCGATCGCCCTTCCCAACAGTTGCG**	Multi-fragment cloning
lacZ-Frag2-Rev2(2388)	**CATGCGGTCGCGTTCGGTTGCACTAC**	
lacZ-Frag3-For3(2362)	**GTAGTGCAACCGAACGCGACCGCATG**	Multi-fragment cloning
lacZ-Frag3-Rev3(3566)	**AACCTCAGCGCGTGTTCTGCTGGCG** ATGGAATAATAGCGAGAACAGAGAAATAG	

Underlined nucleotides indicate the overlapping sequences with the adjacent fragment or vector to be joined.

### Single fragment cloning by Hot Fusion

To assess the efficiency of single fragment cloning, a 3.5 kb DNA fragment containing *lacZ* expression unit encoding β-galactosidase (β-gal) was cloned. The *lacZ* expression unit was amplified from pSV-B-Gal vector by PCR using primers containing from 17 to 20 bp homology to one or the other ends of a SfaAI-linearized binary vector. *LacZ* PCR product was not purified since the template and target vector contained different selectable markers, ampicillin and spectinomycin resistance, respectively. The *lacZ* fragment and SfaAI-linearized vector were added into a PCR tube containing the Hot Fusion master mix and incubated at 50°C for 1 hour as described in the Materials and Methods. Cells were electroporated with 1 µl of the Hot Fusion mix and, after incubation in non-selective medium for 1 hour at 37°C, plated onto LB agar plates containing spectinomycin and X-gal which is a colorimetric indicator of β-gal expression. Thousands of colonies were formed and approximately 95% of the colonies were blue (data not shown) indicating efficient cloning of the *lacZ* gene of about 95%. To confirm directionality and integrity of the junction sequences, four blue colonies were grown in TB containing the appropriate antibiotic and DNA was purified for sequence analysis. Sequencing confirmation determined that all four clones had the expected junctions (data not shown).

### Using Hot Fusion to clone and evaluate plant gene promoters

**Figure 3:**
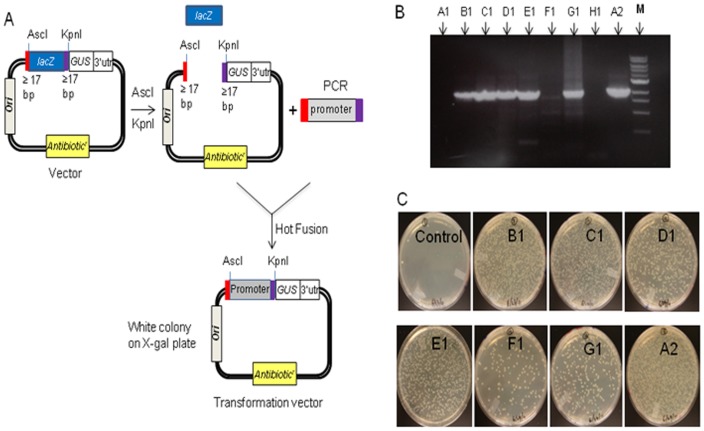
Single fragment cloning by Hot Fusion. (A) A diagram of single fragment cloning in a binary base vector. Base vector containing a *lacZ* gene was linearized by restriction enzyme digestion with AscI and KpnI to release *lacZ*. The linearized vector was directly used for Hot Fusion. (B) Amplified PCR products (A1 through A2) of plant gene promoters were used for cloning. M is a NEB 1 kb DNA ladder. (C) Transformation plates of cloned PCR products (A1 and H1 are not shown). Blue colonies on the plates contain the parental vector and white colonies contain the potential recombinants. Eight white colonies were screened for each construct (Table 2).

Our Gene Expression Technology pipeline investigates the activities of various plant gene promoters. Activity is evaluated by cloning promoters upstream of a β-glucuronidase (GUS) gene lacking its own promoter. To establish an efficient screening system for Hot Fusion cloning, we previously introduced a *lacZ* gene with its own promoter into our binary base vector (over 13 kb) between AscI – KpnI (Acc65I) restriction sites, i.e. the cloning sites where DNA promoter fragments would be introduced (Fig. 3A). For Hot Fusion cloning the base vector was digested with AscI plus KpnI (or Acc65I) to release the *lacZ* gene (Fig. 3A). A PCR fragment containing the promoter to be tested was mixed with the linearized vector and Hot Fusion cloning was carried out. The PCR fragment was designed such that the ends of the fragment overlapped with the ends of the linearized vector by at least 17 nucleotides (Fig. 3A). Since Hot Fusion cloning does not use a ligase, the *lacZ* gene does not ligate back into the base vector, and only the PCR fragment can combine with the vector by homologous base pairing. This creates a unique and automatable HTP system for the selection of positive clones, which represents a significant advantage over systems which use ligase.

To test the Hot Fusion cloning system, nine promoters from corn and soybean gDNA were amplified by PCR. Six promoters gave a high level of PCR DNA fragments whereas three promoters gave little or no PCR fragments (Fig. 3B). The PCR products were directly used for Hot Fusion cloning into the base vector without any purification. Transformation and cloning efficiency are shown in Table 2. The seven promoters with easily detectable levels of PCR products yielded about 200–2000 white colonies (Fig. 3B, C) whereas the two promoters with barely detectable PCR products (Fig. 3B; A1 and H1) yielded only a background level of white colonies (data not shown). Blue colonies on each plate indicated the parental vector that was not completely digested (Fig. 3C). Based on PCR screening of 8 white colonies for each construct, almost all clones had an insert (Table 2), although some inserts were smaller than the expected size (data not shown), which were most likely due to non-specific PCR products amplified (Fig. 3B; F1 and G1). The results show that the cloning efficiency of Hot Fusion is high (at least 80%) when sufficient amounts of PCR inserts are used in the cloning reaction even without purification. It should be noted that the colonies from F1 and G1 plates yielded less than 50% positive clones (Table 2), probably due to the multiple faint PCR products (Fig. 3B, F1) and low-purity of the PCR products (Fig. 3B, G1; smear on the top).

**Table 2:** PCR screening of colonies from nine Hot Fusion transformation plates.

Plate #	PCR product intensity	Total white colonies	Total blue colonies	Colonies screened (white)	Contained insert	Positive clones (with correct insert size)	Efficiency (%)
A1	-	14	4	8	0	0	0
B1	++++	>1,000	3	8	8	8	100
C1	++++	>1,000	5	8	8	8	100
D1	++++	>600	3	8	7	7	88
E1	++++	>800	2	8	8	7	88
F1	+	∼200	3	8	8	3	38
G1	++++	∼250	4	8	8	4	50
H1	-	12	2	8	0	0	0
A2	++++	>1,000	13	8	8	8	100

Eight white colonies were screened for each construct. Non-purified PCR products were used in this study. Efficiency indicates % of clones that contained the expected insert.

### Creation of Seamless constructs

**Figure 4:**
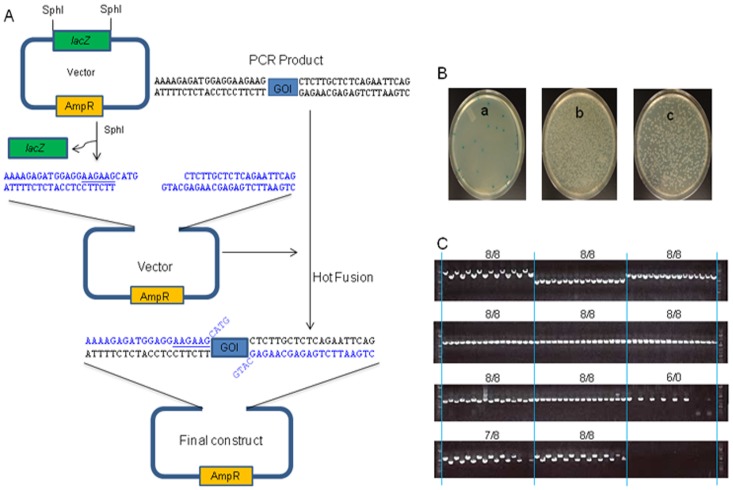
Creation of seamless constructs by Hot Fusion. (A) A schematic diagram for the creation of a seamless construct. The vector was linearized by SphI digestion (leaving CATG 3′ overhangs), heat-inactivated and directly used for cloning. The PCR products contain the overlapping sequences at each end (excluding the CATG overhang). The ribosome binding site is underlined. (B) Examples of the transformation plates. Blue colonies contain the parental vector and white colonies contain potential positive clones. The negative control is shown as plate a (unpurified digested vector without added PCR product). Plates, b and c are two examples of transformation plates containing potential positive clones. (C) PCR screening results of 8 colonies for each construct (each plate). Eight PCR products of each construct were loaded every other lane on agarose gels by multi channel pipette.

There is a need for seamless constructs to minimize any negative effects on gene expression from linker or restriction sites located between a promoter and gene of interest (GOI). Seamless constructs could also increase the efficiency of fusing one coding region with another coding region already present in a vector (such as by removing a stop codon) without the need to re-clone the first coding region. To demonstrate the utility of seamless assembly of constructs by Hot Fusion, we cloned 22 genes encoding various insecticidal proteins into an expression vector. The expression vector contained a 4-bp overhang (CATG) after SphI digestion, which was located between the ribosome binding site and the start codon of genes to be cloned (Fig. 4A). Primers were designed to remove the 4-bp overhang in order for the first ATG of the cloned gene to be closely linked to the ribosome binding site for maximal expression. Following Hot Fusion and electroporation, the control plate produced a small number of blue and white colonies, while the test plates had over a few thousand white colonies representing putative inserts (Fig. 4B). Based on PCR screening of 8 colonies for each construct, 19 out of 22 constructs had 100% inserts (8 out of 8), 2 constructs had 88% inserts (7 out of 8), and only one was negative, yielding an overall efficiency of 94% (Fig. 4C). The negative construct was attributed to the lack of good PCR product.

### Multi-fragment assembly by Hot Fusion

**Figure 5:**
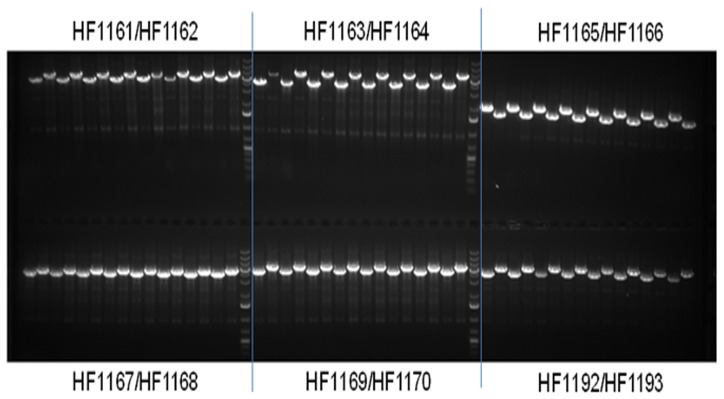
PCR screening of multi-fragment assembled clones from Table 3. Eight clones (colonies) from each construct were grown overnight in the 96-well plate containing LB + Spectinomycin (50 ug/ml). One µl of the over-night grown cell culture was used for PCR screening with base vector primers (18 bp) located immediately upstream of the promoter and downstream of the terminator. PCR products for each construct were loaded every other lane on a 1.0% agarose gel (containing ethidium bromide) with a multi channel pipette. GeneRuler 1 kb Plus DNA Ladder was used as the DNA marker.

One of the challenges in cloning is the ability to rapidly design and construct custom vectors containing different combinations of genes and regulatory elements. The ease of design and the directionality provided by Hot Fusion makes the technique ideally suited for custom cloning applications. To demonstrate the utility of multi-fragment assembly by Hot Fusion, 12 different constructs were designed each consisting of 2–6 kb inserts containing a promoter, GOI and terminator (Table 3). The concentration of purified PCR products ranged from 8–96 ng/µl, and typically 1 µl of each PCR product (without adjusting the DNA concentration) was directly used for Hot Fusion. The vector was linearized by SfaAI cleavage followed by heat-inactivation and was used directly without any purification. While the negative control plate containing only the vector (Table 3; HF1171) yielded only a few dozen blue colonies, as expected, the vector plus three inserts yielded between a few hundred to a couple of thousand white colonies representing putative positive clones (Table 3). Eight clones (colonies) were screened for each construct using base vector primers located upstream from the promoter and downstream from the terminator. Based on PCR screening all clones had the expected inserts (Table 3 and Fig. 5). Similar results were found for a different vector with a different antibiotic selection marker, except that one of the eight clones for one construct had a smaller insert than expected (data not shown), probably due to small non-specific DNA fragment present in the PCR product.

**Table 3:** One step assembly of promoter, GOI and terminator by Hot Fusion reaction.

Hot Fusion	Work plan ID	Insert	Size	Conc. (ng/ul)	Total size (bp)	Blue colonies	White colonies	Colonies screened	Positive colonies
HF1161	CP58017	Promoter	2924	42.3	4615	12	608	8	8
		GOI	1113	42.69					
		Terminator	578	13.92					
HF1162	CP58833	Promoter	2622	20.01	5572	30	1,312	8	8
		GOI	2625	36.96					
		Terminator	325	18.21					
HF1163	CP58837	Promoter	2622	61.27	3978	22	2,576	8	8
		GOI	1031	30.33					
		Terminator	325	15.34					
HF1164	CP59426	Promoter	1940	48.56	5746	26	1,680	8	8
		GOI	3228	26.58					
		Terminator	578	21.58					
HF1165	CP59974	Promoter	941	24.88	2020	42	1,858	8	8
		GOI	579	36.62					
		Terminator	500	33.58					
HF1166	CP59975	Promoter	497	27.18	1657	38	2,976	8	8
		GOI	660	32.27					
		Terminator	500	39.48					
HF1167	CP60007	Promoter	2196	45.27	3874	44	1,940	8	8
		GOI	1428	25.35					
		Terminator	250	25.67					
HF1168	CP60348	Promoter	1583	63.67	4225	32	2,304	8	8
		GOI	2112	29.03					
		Terminator	530	12.18					
HF1169	CP60436	Promoter	2196	31.23	4288	27	1,648	8	8
		GOI	1842	39.75					
		Terminator	250	24.87					
HF1170	CP61067	Promoter	2001	95.99	5055	56	1,360	8	8
		GOI	2804	24.77					
		Terminator	250	20.95					
HF1192	CP61167	Promoter	2196	27.71	3895	22	768	8	8
		GOI	1449	20.79					
		Terminator	250	8.58					
HF1193	CP62253	Promoter	1931	14.45	4634	19	344	8	8
		GOI	2403	44.19					
		Terminator	300	11.7					
HF1171	Control				28	13	NA	NA	NA

A binary vector was digested with SfaAI to release the *lacZ* gene from the cloning site, heat-inactivated and used directly in this study. Blue colonies indicate the parental vector (background) and white colonies indicate potential positive clones. The last column indicates true positive clones based on PCR screening of 8 clones per construct.

### Simultaneous assembly of seven fragments and *in vitro* mutagenesis

**Figure 6:**
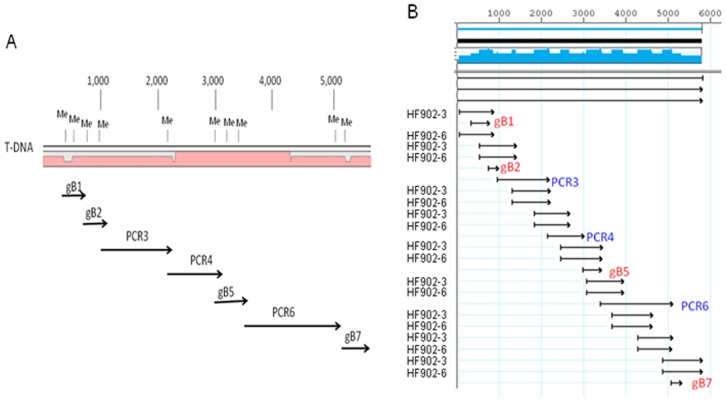
Simultaneous cloning of seven fragments by Hot Fusion in order to eliminate methylation sites. (A) Distribution of *Agrobacterium* methylation sites (ME) on T-DNA of a vector encoding e35s driving the GUS gene. (B) Alignment of two clones (HF902-3 and HF902-6) eliminating ten *Agrobacterium* methylation sites on T-DNA. Four fragments (gB1, gB2, gB5 and gB7) were synthesized to readily mutate the multiple *Agrobacterium* methylation sites within the regions and three fragments (PCR3, PCR4 and PCR6) were amplified from the template with oligo primers containing the mutated base pairs. All seven fragments plus the vector were combined in one Hot Fusion reaction.

To investigate the number of fragments that can be efficiently assembled in one step by Hot Fusion and to demonstrate the utility of Hot Fusion in generating targeted modifications, a 6-kb T-DNA of a binary vector was selected as a target. This 6-kb DNA has 10 potential *Agrobacterium* methylation sites (GANTC) which we wanted to mutate. The sites are unevenly distributed throughout the DNA fragment (Fig. 6A). To knock out these methylation sites, four synthetic fragments and three PCR products were generated by changing one of these five bases (without changing amino acids when the site resided in a coding sequence). The seven fragments were mixed with the recipient vector, which was linearized by restriction enzyme digestion of AscI and HindIII, and the mixture was subjected to a Hot Fusion reaction. The reaction was then transformed into *E. coli* and yielded over a hundred colonies. Eight of these colonies were grown up for DNA isolation. Seven out of eight colonies had the expected restriction pattern (data not shown). Sequencing confirmation determined that all seven plasmids contained the mutated *Agrobacterium* methylation sites, and five contained the expected full length sequences over the entire 6 kb T-DNA region. Alignment of two perfect clones was shown (Fig. 6B). Another two clones contained a single base pair mismatch, possibly due to oligo synthesis errors since one mismatch was in the oligo primer sequence used for PCR and the other was in a synthesized fragment (gBlocks) provided by the vendor. Therefore, it is highly unlikely that the mutations were introduced as a result of a Hot Fusion process. Given that five of the eight plasmids were base perfect, this should not be a major concern with the appropriate DNA sequencing confirmation as quality control.

### RNAi constructs

**Figure 7:**
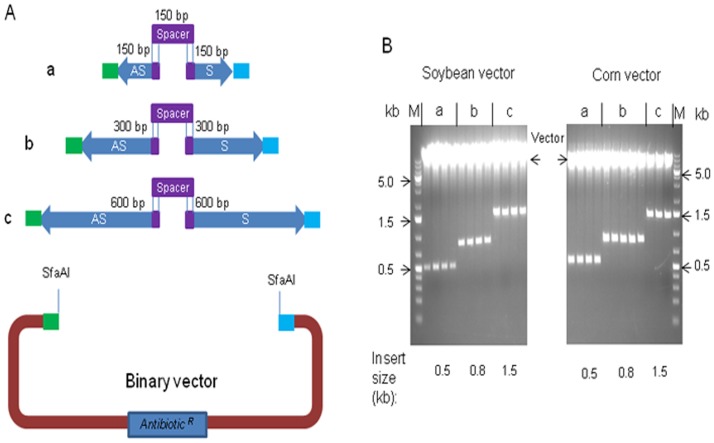
Creation of RNAi constructs by Hot Fusion. (A) Design of RNAi elements for Hot Fusion. Anti-sense (AS) and sense (S) fragments of 150 bp (a), 300 bp (b) and 600 bp (c) overlapped with the vector and spacer as indicated. (B) Restriction digestion of plasmids containing RNAi elements. Three to five clones were analyzed for each construct (a, b and c). Each clone was digested by AscI and PmeI flanking the insert. The a, b and c contained the 150 bp, 300 bp and 600 bp of both anti-sense and sense fragments, respectively. M represents GeneRuler 1 kb Plus DNA Ladder.

It has always been a challenge to make RNAi constructs due to inverted repeats, which readily form secondary structures and inhibit DNA synthesis by DNA polymerase. With Hot Fusion the inverted repeat could be synthesized or amplified by PCR as two separate parts, forward-sense and reverse-antisense. In this study sense and anti-sense DNA fragments of 150 bp, 300 bp and 600 bp in length were amplified from DNA templates through PCR. A 150 bp spacer was synthesized through gBlocks to join the ends of the sense and antisense fragments. Approximately 30 bp at either end of the spacer was homologous to corresponding regions on the sense and anti-sense fragments so that the three fragments could anneal together (Fig. 7A). The sense, antisense and spacer fragments were mixed with a restriction-digested base vector (corn or soybean vector) and the mixture was subjected to the Hot Fusion reaction. Subsequently the Hot Fusion mix was transformed into *E. coli* cells which showed that at least 95% of the colonies were white indicating that the vector had taken up a DNA fragment. Plasmid preps were done on three to five colonies for each class of insert size. Digestion showed that each plasmid contained the expected size of inverted repeat plus spacer (Fig. 7B). Sequencing confirmed that these clones had the correct assembly (data not shown). These results demonstrate that Hot Fusion is an efficient method for making RNAi constructs and also indicate that the 150 bp spacer was long enough for the Hot Fusion reaction at the conditions used.

An alternative experiment with a 30 bp spacer was also carried out. The 30 bp spacer was attached to both the 3′ of anti-sense fragment (600 bp, 300 bp, 150 bp) and 5′ of the sense fragment (600 bp, 300 bp and 150 bp) as the overlapping sequences. It was expected that the two fragments would anneal in the Hot Fusion reaction at their common 30 bp spacer. The two fragments were mixed with a restriction-digested base vector and the mixture subjected to a Hot Fusion reaction. Transformation of the reaction into *E. coli* cells followed by plasmid analysis of transformed colonies showed that the two-fragment method of producing RNAi inverted repeats worked as well as the former method using three fragments (sense, antisense and spacer) (data not shown).

### Comparison of Hot Fusion with NEB Gibson Assembly cloning kit

**Figure 8:**
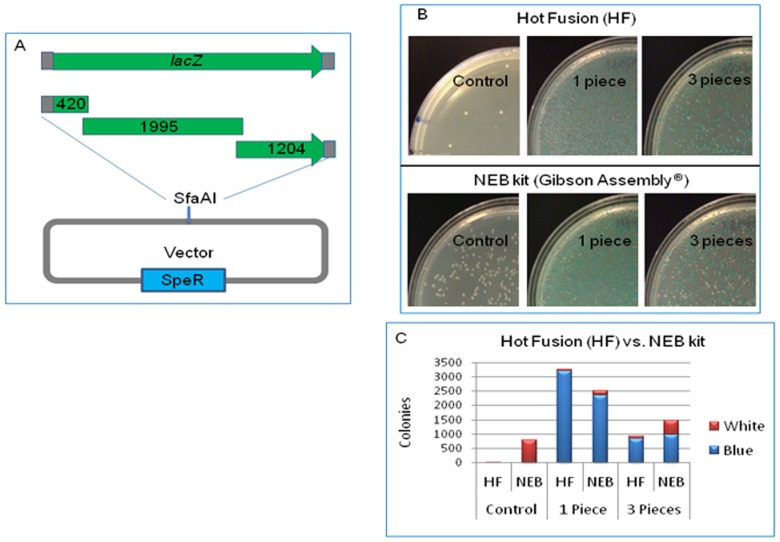
Comparison study of Hot Fusion and Gibson Assembly (NEB kit). (A) Cloning strategy for single and multi-fragment assembly. A full *lacZ* gene and three pieces of the *lacZ gene* with overlapping sequences were amplified, and cloned into a base vector digested with SfaAI (unpurified). (B) Transformation efficiency of *lacZ* assembly by Hot Fusion and Gibson Assembly (from NEB). The top panels are the transformation plates from Hot Fusion. The bottom panels are the transformation plates from Gibson Assembly. The same amount of base vector was used in all reactions and the same amount of insert was used in the same treatment. Blue colonies contain the *lacZ* gene or all three pieces of the *lacZ* gene correctly cloned or assembled together (in frame). White colonies contain the parent vector. (C) Cloning efficiency of Hot Fusion and Gibson Assembly. Colonies were counted from each transformation plate.

Recently a commercial Kit (NEB, MA) based on Gibson assembly has become available. The Gibson Assembly cloning kit utilizes three key enzymes, T5 exonuclease, Phusion DNA polymerase and Taq DNA ligase. To test whether the NEB kit has a better cloning efficiency (since it contains Taq ligase) than Hot Fusion, single and multi-fragment assembly of *lacZ* were conducted using both NEB kit and Hot Fusion, respectively, with a single enzyme-digested base vector (unpurified) (Fig. 8A). It was observed that Gibson Assembly yielded over 10 fold more background colonies than Hot Fusion (a few hundred vs. a few dozens) (Fig. 8B; the control plates), apparently due to the self-ligation of the vector by Taq ligase. It is noteworthy to mention that for single fragment cloning both Gibson assembly and Hot Fusion had similar cloning efficiencies (98% vs. 99% in this case) and yielded over a thousand positive clones (Fig. 8B, C). For multi-fragment assembly however, the cloning efficiency of Gibson assembly dropped to 70% due to more background colonies produced by self-ligation (Fig. 8B, C), whereas Hot Fusion reached 92% efficiency (Fig. 8B, C). These results indicate that Hot Fusion actually has an advantage over NEB kit (Gibson Assembly), and that the absence of Taq DNA ligase significantly lowers the self-ligation of base vectors without decreasing the overall cloning efficiency. Neither alkaline phosphatase treatment (for single enzyme-digested vector) nor purification of base vectors (for two enzyme-digested vectors) is needed and thus these extra steps can be omitted for Hot Fusion, which is more amenable for automation.

### Ten minutes Hot Fusion reaction

**Figure 9:**
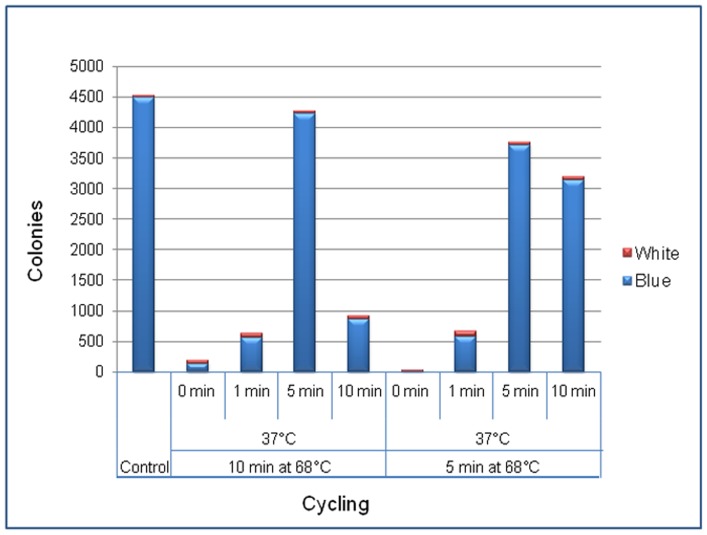
Comparison study of Hot Fusion under different cycling conditions. A full lacZ *gene* with overlapping sequences to a vector was amplified and used for testing. The same amount of linearized base vector and *lacZ* insert were used in each reaction. The vector and insert were mixed and incubated at 37°C for 0, 1, 5 or 10 minutes, respectively, and then switched to 68°C for 5 or 10 minutes, respectively. The control used the regular Hot Fusion condition (1-hour at 50°C) as described in the Materials and Methods. Blue and red bars indicate numbers of clones containing either the *lacZ* gene or empty vector, respectively.

To investigate a minimum time needed for Hot Fusion reaction, a number of conditions were tested with optimal temperature for T5 exonuclease and Phusion Hot Start DNA polymerase. We found that a 10-minute Hot Fusion reaction was sufficiently enough for single fragment cloning, which was simplified by incubation of the tube at 37°C for 5 minutes and 5 minutes at 68°C (Fig. 9). However, for assembly of multiple fragments the best results were still observed using the regular conditions (1-hour at 50°C) as described in the Materials and Methods (data not shown).

## Discussion

Due to the demand of large quantity and complexity of constructs for crop and non-crop pipelines, a high throughput method termed Hot Fusion that is simple, robust, efficient and automation friendly has been developed. The method is especially useful for assembly of multiple pieces and RNAi constructs.

Hot Fusion is based on the requirement that two or multiple fragments (including the destination vector) share 17 bp–30 bp overlapping sequences with the adjacent fragment to be joined. The cloning is simply carried out in a one-step reaction at 50°C for one hour. The reaction buffer contains two key components: T5 exonuclease and Phusion DNA polymerase. T5 exonuclease removes nucleotides from the 5′ to 3′ end leaving single strand tails of 3′ overhangs that are complementary and anneal to single strand tails of the adjacent fragment or vector generated by the T5 exonuclease in the same reaction. Phusion DNA polymerase fills in gaps that are over-generated by T5 exonuclease. Annealed fragments are transformed into *E. coli* and the nick sites in the nucleotide chain are repaired by *E. coli.* This is in contrast to the NEB kit for Gibson Assembly which contains the additional enzyme of Taq ligase to seal gaps generated byT5 exonuclease *in vitro*. In our experience 17-bp overlap sequences were sufficient for single fragment cloning, but 25 bp–30 bp overlapping sequences were ideal for multiple fragment assembly. In addition, these short overlapping sequences plus element-specific sequences are usually less than 60 bp, which allows for the fast delivery of the oligos with lower cost.

To establish an efficient screening process for positive clones (i.e., clones with inserts), we first introduced *lacZ* into our binary base vectors used routinely for cloning. Introduction of *lacZ* into base vectors provided a quick, easy and visual quality check for vector preps and allowed visual selection of potential positive clones as the uncut vector will be blue on an X-gal plate, while potential positive clones will be white. This eliminated the need for purification of digested vectors which is often a source of significant loss of vector DNA, particularly for large vectors. It therefore provided a counter screen for positive clones and minimized PCR screening, thus resulting in significant reagent and time savings.

Hot Fusion has worked very efficiently for single fragment cloning by using both *lacZ* gene and PCR products amplified from cDNA, gDNA and plasmid DNA templates. Based on cloning of over a thousand genes and elements (i.e. promoters), the efficiency reached over 95% (with expected inserts) when good quality PCR products were used. Sequence analysis of hundreds of clones, showed that approximately 90% of the clones were perfect in both the overlapping regions and cloned inserts, although we did see some mutations (mismatches or deletions) occurring in some individual clones that could have been due to PCR or the unpurified oligos used for PCR. It is noteworthy to mention that Hot Fusion was very forgiving to the amount of insert DNA used (from 10 ng to 100 ng), compared to other methods like Cold Fusion (data not shown). For synthesized fragments (i.e. gBlocks from IDT), Hot Fusion worked well even with 2–10 ng of insert DNA, probably due to the high purity of the synthesized DNA compared with gel-purified PCR products. In some cases it was helpful to gel-purify the desired PCR product from non-specific products in order to reduce the number of unwanted clones. In addition, smaller vectors (4–6 kb) appeared to work more efficiently than larger vectors (>12 kb), yielding more colonies (data not shown).

To investigate whether seamless constructs could be made by Hot Fusion an insect control discovery expression vector was initially used for testing. A vector containing *lacZ* for blue/white selection was digested by SphI to excise *lacZ* and the vector used directly for cloning GOIs by Hot Fusion (Fig. 4). Oligos used to amplify GOIs excluded the 4-bp overhangs (CATG) produced by SphI in order to keep GOIs close to the ribosome binding site (AAGAAG) for maximal expression of GOI. Based on transformation, and PCR screening and sequencing confirmation, seamless constructs were efficiently constructed by Hot Fusion, and reached over 94% efficiency (based on construction of a few hundred constructs). In other experiments as many as 37 bp were intentionally removed between overlapping regions by Hot Fusion cloning (data not shown), but generally cloning efficiency was higher when fewer base pairs were intentionally removed between overlapping regions. Overall the results indicate that Hot Fusion is an efficient and useful method to make junctions with exact desired sequences between fragments.

Notably Hot Fusion also worked well for multi-fragment assembly. To investigate how efficient Hot Fusion works for multi-fragment assembly, 12 nominations of custom constructs with different promoter/GOI/3′UTRs combinations were tested in a high throughput manner (without adjusting concentrations of each PCR-amplified DNA fragment). The cloning efficiency reached 99–100% for three fragment (insert) assembly in this case (Table 3), and an overall pass rate of 85–95% was observed for three fragment assembly (base perfect clones), dependent on the size of fragments used. It appears that the smaller the total sizes of the assembled inserts and the fewer the fragments to be assembled, the more efficient assembly by Hot Fusion is. It is noteworthy to mention that optimization of DNA to vector ratio or adjusting DNA concentration was not necessary in most cases, particularly for single or two fragment cloning (with a range of 20 ng to 100 ng). However the efficiency could be enhanced with a vector to insert ratio of 3∶1 (data not shown). An excess of inserts (i.e., 10 fold), particularly for more than three fragment assembly didn't help for the efficiency, instead yielded fewer colonies probably due to competition of each DNA molecule for enzymes. This could cause imperfect chew-backs for each molecule at both 5′ and 3′ ends (due to enzyme limitations) (data not shown). Seven fragments (more than 6 kb) were successfully assembled by Hot Fusion in one step, indicating that Hot Fusion is an efficient method to build complex constructs or entire vectors. In fact, we constructed a number of large vectors (a total size of 13–15 kb) through assembly of eight fragments at once by this method (data not shown). Combination of synthesized fragments and PCR products (Fig. 6) could become a useful approach in site-directed mutagenesis and complex constructs if a gene or element can not be amplified or synthesized due to high GC content or strong hairpins.

Although UDG-based LIC is an efficient method for making RNAi constructs [17], it has some limitations. It requires two successive rounds of PCR to generate the final PCR product used for cloning. Particularly due to the nature of the Expand High Fidelity^PLUS^ DNA polymerase used in the 2 ^nd^ PCR, the amplification is often not robust. Remediation has to be conducted for the 2^nd^ PCR for many clones. In addition cloning of inverted repeats also involves two step reactions [17]. Type IIs-based RNAi construction was also recently reported [27], but is not suited for large binary vectors. To investigate whether Hot Fusion works for RNAi constructs, different lengths of inverted repeats were used for testing. All these inverted repeats were successfully assembled in one step by Hot Fusion and the 150 bp spacer used for RNAi constructs was stable in the Hot Fusion reaction. The results indicate that Hot Fusion is an efficient method to build RNAi construct in a single step.

Recently Gibson Assembly has been broadly used for assembling large genomes and cloning. Gibson Assembly has the additional enzyme of Taq ligase that seals gaps generated by T5 exonuclease *in vitro*. To compare the efficiency of Hot Fusion with Gibson Assembly a single enzyme (SfaAI)-digested (and non-purified) vector was used for cloning of single and three fragments, respectively. It was observed that the Gibson Assembly method yielded many more non-recombinant background colonies than Hot Fusion. Although cloning efficiency for Gibson Assembly was not significantly affected with single fragment cloning, the efficiency dropped considerably for three fragment assembly when compared to Hot Fusion. This decrease in efficiency may have been due to self-ligation of the vector by Taq ligase used in the kit. Alkaline phosphatase (CIP) - treated vector could reduce some background, but may be inconvenient because it introduces an extra step in the cloning process. Therefore, Hot Fusion has advantages over Gibson Assembly because Hot Fusion does not cause self-ligation, does not need CIP, nor does it require any purification of enzyme-digested vectors. The Hot Fusion method is well suited for high throughput applications that require a simple, robust, efficient and automatable process for single and multiple fragment assembly in a one step cloning process. Single fragment cloning can even be done in a 10-min reaction. The primer design is also simple and is restriction site independent.
